# Metacommentary: Identifying and Mastering ‘Dear Reader’ Moments

**DOI:** 10.5334/pme.891

**Published:** 2023-02-20

**Authors:** Lorelei Lingard

**Affiliations:** 1 Western University, CA

## Abstract

You know those worry spots in your paper, the ones you know are there but you’re sort of hoping the reader won’t notice? They will. Whatever the worry spot, big or small, it needs attention before you consider the paper ready for submission. This Writer’s Craft treats these worry spots as “Dear Reader” moments – that is, moments where you should directly address a potential reader concern – and explains how to use metacommentary to achieve this.

“You’re writing two texts joined at the hip: one makes the argument, the other ensures your argument isn’t mistaken for one you don’t want to make.”

(Graff & Birkenstein, 2014) [1]

## Who is your reader?

Approaching your writing as a conversation between you and your reader is a sure way to make your publications more engaging and persuasive. There are two aspects to this approach: first, think about who the journal’s general readership is, and then identify among that readership your specific target reader. Many of us, for better or worse, write with our peer reviewers in mind, imagining the holes they might poke in our science and our story. But “peer reviewer” is too generic a reader: we need to be more precise. Are your target readers opinion leaders in your field, experts from adjacent fields, informed laypeople, or representatives of the other side of a controversy? Once you start to think this way, you will realize that different readers require different strategies to keep them on track. For instance, imagine you are writing about the role and impact of clinical competency committees and your first sentence is this:

Competency-based medical education, with its emphasis on learners’ achievement of patient-centered outcomes during training, demands a rethink of long-standing conventions in our field.

If your intended audience is a naïve reader, the metacommentary in this sentence serves to clarify what’s meant by competency-based medical education (CBME). For a reader already engaged in the CBME conversation, the metacommentary might seem unnecessary. However, for a reader who takes an opposing view in the CBME conversation, the metacommentary might come across as manipulative because it presents a matter-of-fact claim that CBME emphasizes patient-centred outcomes. Clarification is the wrong meta-move for the opposed reader. Instead, you need anticipation and deflection:

Competency-based medical education, regardless of one’s stance in the current debate regarding its use, demands a rethink of long-standing conventions in our field.

Decide who your main audience is and write to keep them with you as the manuscript unfolds. You can nod to other readers periodically, for instance by explaining theories that the general reader might not recognize, but keep your primary conversant in the foreground.

## What is a worry spot?

Now that you have your primary reader in mind, be alert to the places in your manuscript where you’re worried they might not come along with you. Imagine that you’re talking across a table: ‘worry spots’ are the moments when they might interrupt, or stop nodding, or begin to scowl. Maybe you defined your terms in an unusual way; yes, the reader will quibble. Maybe you used a hybrid methodology; yes, the reader could prefer the purist version. Maybe you settled firmly on one side of a controversy; yes, the reader may sit on the other. What we’re most interested in are worry spots at fundamental points in your paper: if you don’t deal with these worry spots, readers may misunderstand your key terms, mistrust your methods, reject your main premise or resist your argument.

You might be thinking, why go looking for worry spots? After all, peer review is going to point them out, right? Indeed, it will! And anyone who has prepared a revised manuscript and response to reviews has produced metacommentary. However, this is metacommentary *after the fact*. And it may be too late, because once the reader encounters a worry spot, their impression of the manuscript could be colored from that point on. Let’s say you purposefully left a theory out of your conceptual framing; if the reader assumes that you don’t know the theory (rather than understanding that you judged it less relevant), they may read everything that follows prejudicially. It’s not that readers are contrary and resistant, looking for any reason to downgrade your work. Honestly, most of them are not. But if they experience a number of worry spots left untreated, they will hit a tipping point where they shift from a tolerant reader to a resistant one. Imagine the reader, proceeding through a manuscript, who is thinking: What do they mean by that term? Aren’t they aware of the history of this concept? Why on earth did they choose survey methods? Why isn’t the discussion more theoretically grounded? You can see how such worry spots can accumulate and color their impression of the work. By predicting when they will arise for the reader and proactively addressing them with *just in time* metacommentary, writers can manage reader impressions before this shift occurs.

## What is metacommentary?

Once you’ve identified the worry spots, you want to turn these into “Dear Reader” moments. You do this by inserting metacommentary that addresses the worry directly or indirectly. For instance, if you are worried that your introduction might alienate nonexpert readers, you might write the following metacommentary to clarify your terms:

Clinical training, based on an apprenticeship model in which medical learners of various levels do clinical work alongside and supervised by medical practitioners with more experience, serves both educational and service purposes.

This metacommentary effectively says, “Dear Reader, let us clarify what we mean by the term ‘clinical training’”. However, if your readers are clinical educators rather than nonexperts in the field, you won’t need the clarifying metacommentary because they already know what ‘clinical training’ means. You’re probably safe to simply write: “Clinical training serves both educational and service purposes.” Knowing your audience is key to getting metacommentary right.

Metacommentary is one of the techniques writers use to keep the reader on track. It sits alongside your main text, guiding interpretation: as Graff and Birkenstein explain, “the main text says something, the metatext tells readers how – and how not – to think about it” (G and B 130). They liken metacommentary to a voice-over narrator in a TV show or the chorus in a Greek play, explaining to the audience what’s happening as the scene unfolds. Table 1 illustrates some common worry spots that writers might identify, offers examples of metacommentary to insert in the manuscript to address these, and explicitly *translates* the ‘Dear Reader’ voice-over.

**Table 1:** Common Worry Spots, Metacommentary, and ‘Dear Reader’ Voice-Over, adapted from Lingard & Watling 2021 [2].


I’M WORRIED THAT…	METACOMMENTARY	‘DEAR READER’ VOICE-OVER

My terms won’t be clear	In using the term “decolonization”, we intend…	*Dear Reader, let us explain our terminology*.

My methodological choices will be critiqued	Interview data is perceptual and subjective, but this is a strength rather than a weakness. For if learners perceive mistreatment, then they subjectively experience mistreatment.	*Dear Reader, you may be worried about the limits of interview data. Let us elaborate*.

The lineage of my work won’t be recognized	Following work from sociolinguistics, we treat voice as socially constructed and negotiated…	*Dear Reader, our approach is based on other scholars who have come before and whom you may recognize as credible*.

My awareness of related work will be questioned	While other fields have made recent advances in the science of big data, medical education…	*Dear Reader, in case you were wondering whether we are aware of scholarship in other fields, we reference it here*.

There will be resistance to my ideas or methods	While qualitative researchers debate the notion of ‘emergence’, we use this term aware that…	*Dear Reader, we recognize that there is more than one way to approach this issue*.

Those who disagree with me will dismiss my work	Although it has been argued that there are no low stakes assessments, we contend …	*Dear Reader, we know there is controversy in the field, and we align ourselves consciously*.


Metacommentary is often aimed at anticipating objections: of the examples in Table 1, all but the first would fall into this category. This is a delicate dance, because your goal in anticipating the objection is to ease, not intensify it. Consider this example: “Our network analysis may be viewed as superficial, given that it included only authors of records selected for our review, not authors of references cited in those records.” By using the term “superficial”, this metacommentary risks naming that concern into existence in the reader’s mind. More effective might be: “Our network analysis was limited to authors of records included in our review, and excluded authors of references cited in those records.” Notice that the second example is equally direct about the concern – a limited network analysis – but avoids escalating that concern through a term like “superficial”. Graff and Birkenstein warn that “when you write objections into a text, you take the risk that readers will find those objections more convincing than the argument you yourself are advancing” (p 87). So anticipate and represent objections fairly, but don’t give them more persuasive power than you intend.

## Recognizing metacommentary at work

Now that you’re attuned to metacommentary as ‘Dear Reader’ moments, try to notice these in others’ writing. This is a good way to increase both your awareness of what might constitute a worry spot and your repertoire of metacommentary to respond. You can always find them in the Limitations section of a paper, but we’re more interested in *just in time* metacommentary that happens before that formal, and usually late, section. For instance, Wyatt et al are dealing with a ‘Dear Reader’ moment related to theoretical positioning when their introduction acknowledges that [3]:

Although the USA is technically not a post-colonial society, these barriers are common among societies with colonial roots.^10, 11^ For this reason, social scientists working outside medical education frequently employ post-colonial theory^10, 12^ as a research orientation to support minoritised populations in the USA.^13–15^

What’s their worry? It seems to be that readers familiar with post-colonial work might take issue with this use of ‘post-colonial theory’. The authors’ voice-over says, “Dear Reader, we know our study context is not technically ‘post-colonial’, but we follow other scholars in using this theory in this domain and justify it in terms of our focus on minoritized populations”.

Similarly Kinnear et al, [4] wading into the validity conversation, position themselves carefully within a conceptual debate in their introduction:

Two contemporary frameworks are commonly used to organize validity evidence, though they differ in emphasis (Table 1). Messick’s framework stresses *sources* of validity evidence,16 and Kane’s focuses on *inferences* in an evidentiary chain.17 While these frameworks are not mutually exclusive, often one or the other is used to organize evidence. However, we believe they are complementary. Different sources of evidence (Messick) can support argument inferences (Kane) and provide a more complete picture of a complex validity argument.

The worry here seems to be that readers may find their use of both validity frameworks, rather than one or the other, imprecise. Their voice-over says, “Dear Reader, we know these are two (often separate) camps and it’s a bit sacrilegious to use them complementarily, but we do so consciously and carefully.”

Methods sections are also a good place for metacommentary. Chan et al [5] use metacommentary to address worries about their sample size:

We used a random number generator to number the database items, and then used the first 50 comments. As no previous work on this score has been completed, we were unsure about our sample size calculation, and therefore selected 50 comments to ensure that we had adequate ratings to conduct our analyses.

The Dear Reader voice-over here says, “We know that sample size calculation is imprecise, so we purposefully over sampled.” This is a strategic place for this acknowledgement of a concern potentially lurking in the reader’s mind: why wait until the Limitations to address such a central issue?

Another important place for metacommentary is in the body of the writer’s argument. For instance, LaDonna et al [6] seem to recognize that anyone who sets out to “quantify qualitative rigor” is in for resistance:

While we hesitate to quantify qualitative rigor, we suggest that interview length may be a more useful indicator of information power than sample size. While this guidance is not foolproof and should not be followed prescriptively….

Their Dear Reader commentary acknowledges that they could be treading on shaky ground with qualitative readers: they “hesitate”, and explicitly acknowledge that their guidance “is not foolproof” nor intended to be prescriptive.

Perhaps most important is metacommentary related to the fundamental premises of a paper, because if the reader resists you in these moments, the rest of the piece may not matter. Barrere-Cain et al [7] recognize this in a recent argument to include civic health advocacy in medical school curricula:

While some argue that civic health advocacy is outside the purview of the medical profession,14 these efforts are not dissimilar to other physician activist movements such as White Coats for Black Lives and American Foundation for Firearm Injury Reduction in Medicine (AFFIRM) that have gained popular support.15–1

The worry that readers might disagree with their call for curricular reform is acknowledged explicitly and handled persuasively by aligning the cause of civic health advocacy with other popular movements. The voice-over here is “if you recognize and support these other physician activist movements, then you should also recognize and support this one”.

## Trying out metacommentary in your writing

If metacommentary feels a bit like a foreign language for you, you can use Graff and Birkenstein’s templates to practice. They offer templates for common situations (Table 2).

**Table 2:** Adaptation of Graff and Birkenstein’s metacommentary templates.


METACOMMENTARY MOVE	TEMPLATES

To ward off potential misunderstandings	Our argument is not that ----, but that ----- We do not intend ---- To be clear, ----

To elaborate on a previous idea	In other words, ---- This could mean that ---- To elaborate, ----

To provide a roadmap to the text	Having established that ----, we will now consider ---- Our results are presented from most to least prominent -----

To move from general claim to specific example	As a case in point, consider ---- For example, ----

To indicate the relative importance of claims	More importantly, ---- Of less significance, ---- Incidentally, we would note that ----

To make concessions while standing your ground	Although we recognize that -----, we nevertheless maintain that ---- These scholars have a point; however we----

To represent and answer objections	Our approach is unconventional, but necessarily responsive to ---- Some may disagree with this framing; however, it allows us to ----


As you play with such templates, remember that metacommentary can be tricky. Sometimes our metacommentary doesn’t actually say what we intend it to. Consider this sentence, written to make concessions while standing ground:

Systematic reviews are the gold standard for evaluating knowledge; however, we opted to conduct a scoping review.

This sentence makes a concession, admitting that the authors have selected a methodology other than the gold standard: it does not, however, do a good job of standing ground. If we translated it into a Dear Reader voice-over, it would say something like: “We know systematic reviews are the best, but we decided to do something else.” This metacommentary would be made more effective with a revision like:

While systematic reviews are the gold standard for evaluating knowledge, they require a robust body of evidence which our literature of interest does not provide; therefore, given the limited literature available, we opted to conduct a scoping review.

The concession remains the same, but the decision to do a scoping review is justified with reference to the requirements for systematic reviews, thus more persuasively standing ground.

As this example illustrates, vocalizing the “Dear Reader’ translation can help you to make sure your metacommentary says what you want it to.

## In summary

There is no such thing as a manuscript without worry spots. And, yes, you could use your Limitations section to reflect on them, or you could wait to see which worry spots reviewers notice and address those following peer review. But a more effective strategy is to deal with your worry spots proactively, to avoid having them flavor the reader’s interpretation and impression of your work from the moment they appear. Learn to use *just in time* metacommentary and you will be able to talk your readers through the worry spots, keeping them with you as your story unfolds.

## References

[1] Graff G, Birkenstein C. They Say/I Say: The moves that matter in academic writing. New York: WW Norton & Company; 2006.

[2] Lingard L, Watling C. Story, not Study: 30 brief lessons to inspire health researchers as writers. Springer Nature; 2021. DOI: 10.1007/978-3-030-71363-8

[3] Wyatt TR, Balmer D, Rockich-Winston N, Chow CJ, Richards J, Zaidi Z. ‘Whispers and shadows’: A critical review of the professional identity literature with respect to minority physicians. Med Educ. 2021; 55: 148–158. DOI: 10.1111/medu.14295 33448459

[4] Benjamin K, Matthew K, Brian M, Dana S, Daniel SP, Daniel SJ, Eric WJ. Constructing a Validity Map for a Workplace-Based Assessment System: Cross-Walking Messick and Kane. Academic Medicine. July 2021; 96(7): S64–S69. DOI: 10.1097/ACM.0000000000004112 34183604

[5] Chan Sebok-Syer SS, Sampson C, Monteiro S. The Quality of Assessment of Learning (Qual) Score: Validity Evidence for a Scoring System Aimed at Rating Short, Workplace-Based Comments on Trainee Performance. Teaching and Learning in Medicine. 2020; 32(3): 319–329. DOI: 10.1080/10401334.2019.1708365 32013584

[6] LaDonna KA, Artino AR, Balmer DF. Beyond the Guise of Saturation: Rigor and Qualitative Interview Data. J Grad Med Educ. 1 October 2021; 13(5): 607–611. DOI: 10.4300/JGME-D-21-00752.1 34721785 PMC8527935

[7] Rio B-C, Meera G, Dahlia KA, Carlton L, Alexander R, Alister M. Why and How Civic Health Should Be Incorporated into Medical Education. Academic Medicine. December 2022; 97(12): 1760–1764. DOI: 10.1097/ACM.0000000000004765 36149387

